# Dr. Thomas E. Smyth

**Published:** 1910-06-18

**Authors:** 


Dr. Thomas E. Smyth has died at Tavistock in his 47th year. He studied at Dublin, taking his M.B. and B.Ch. in 1886, and graduated M.D. in 1890. He gained a special certificate in gynæcology at the Dublin Rotunda Hospital. His appointments included that of house physician and house surgeon at the Royal City of Dublin Hospital; Government Medical Officer, Campbelltown, N.S.W.; and physician and surgeon to the Tavistock Hospital. A few contributions of his appeared in the *Australian Medical Gazette.*

